# Rhabdomyosarcoma debut masquerading as acute lymphoblastic leukemia: A case report and review of the literature

**DOI:** 10.1002/ccr3.2284

**Published:** 2019-07-02

**Authors:** Bernardo López‐Andrade, Maria Antonia Duran, Lourdes Torres, Marta García‐Recio, Laura Lo Riso, Alejandro Formica, Rafael F. Ramos, Niurka Cerda, Antonia Sampol

**Affiliations:** ^1^ Servicio Hematología y Hemoterapia Hospital Universitario Son Espases Palma Mallorca Spain; ^2^ Servicio Hematología y Hemoterapia Hospital Can Mises Ibiza Spain; ^3^ Servicio Anatomía patológica Hospital Universitario Son Espases Palma Mallorca Spain; ^4^ Servicio Anatomía patológica Hospital Can Mises Ibiza Spain

**Keywords:** hemophagocytosis, leukemia, leukemic presentation, rhabdomyosarcoma

## Abstract

Bone marrow infiltration by alveolar rhabdomyosarcoma is uncommon, some cases can mimicry acute leukemia at presentation and mislead the diagnosis. The integration of diagnostics tests and techniques in uncommon malignancies is important to suspect and reach the diagnosis and avoid delay on treatment. We report a case of alveolar rhabdomyosarcoma bone marrow infiltration associated with hemophagocytosis and cell cannibalism, mimicking acute leukemia at presentation.

## 1 INTRODUCTION

Pancytopenia secondary to bone marrow infiltration is often found at the diagnosis of hematologic neoplasms. However, other neoplasms can have a similar presentation, and, in the presence of a dry tap bone marrow aspiration, the diagnosis can be delayed. We report a case of bone marrow infiltration by a nonhematologic neoplasm associated with hemophagocytosis and cell cannibalism, mimicking acute leukemia at presentation. Alveolar rhabdomyosarcoma is uncommon in adults, even more the bone marrow infiltration by this neoplasm, we review previous cases reported in the literature.

## 2 CASE REPORT

A 56‐year‐old woman tourist with no previous medical history was admitted for persistent back pain and epistaxis to a partner center. The complete blood count reported pancytopenia with Hb 3.6 gr/dL, MCV 101 fl, platelet 10 000 10^3^/µL, and absolute neutrophil count of 0.960 10^3^/µL. coagulation test, C‐reactive protein, and Coombs tests were normal, and an increased lactate dehydrogenase 3344 U/L was reported with no other biochemistry alterations. The peripheral blood smear confirmed the pancytopenia with no further findings (no tear cells, erythroblasts, or abnormal white blood cells were reported.). A CT scan of the spine reported a fracture of the D10 vertebral body with a perivertebral soft tissue component. Initially, biopsy was postponed due to severe thrombocytopenia. On suspecting a hematological neoplasm, a bone marrow aspiration was performed obtaining a dry tap, which was followed by a bone marrow biopsy, where a bone marrow touch imprint of the cylinder was performed. The patient was referred to our center under suspicion of acute lymphoblastic leukemia due to the presence of basophilic blast‐like cells in the imprint and the high requirement of blood transfusion.

On arrival at our center, a new bone marrow aspirate was performed obtaining a sample of bone marrow blood just enough for Immunophenotyping. We proceeded to make a detailed review of the samples sent with the patient.

**Figure 1:**
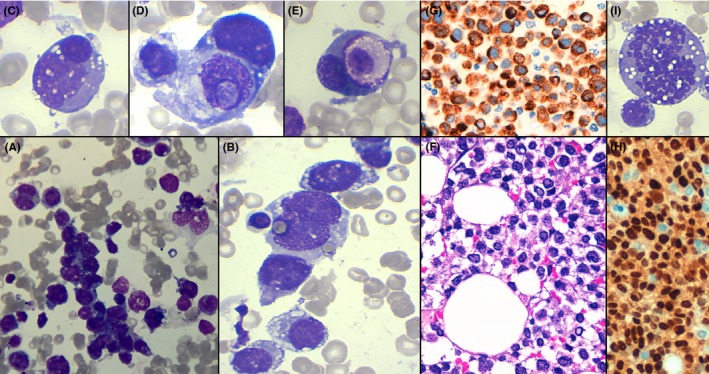
May‐Grünwald‐Giemsa bone marrow imprint 50× oil objective (A); 100× oil objective (B, C, D, E, I). Bone marrow trephine biopsy 20× objective (F). Bone marrow desmine 40× objective (G). myogenin 40× objective (H)

The Grunwald‐Giemsa bone marrow imprint revealed the presence of a bone marrow infiltration by large cells with a round nucleus with lax chromatin and basophilic cytoplasm with vacuoles, with some semblance to lymphoblast morphology. The cells showed a tendency to coalesce into groups Figure 1A, which is rarely seen in hematopoietic neoplasm.

Hemophagocytosis Figure 1B and cell cannibalism of the abnormal cells and other peripheral blood cells (Figure 1C‐E) were observed. At this point, we decided to wait for further results to discard other neoplasms. The Bone marrow immunophenotype was able to confirm the presence of 90% large cells CD45−, CD56+, and incompatible with hematopoietic cells, which oriented the diagnosis to a nonhematological neoplasm infiltration.

Bone marrow trephine biopsy confirmed the diagnosis, observing at 75× infiltration of large mononucleated cells Figure 1F. The immunohistochemistry found a high expression of desmine Figure 1G and myogenin Figure 1H, which was compatible with the diagnosis of bone marrow infiltration by alveolar rhabdomyosarcoma. Due to the tissue preparation, it was impossible to perform a cytogenetic test in trephine biopsy in order to search for t(2;13)(q35;q14).

The primary tumor was suspected to be the soft tissue mass englobing D10 reported in the CT scan. Nevertheless, we were unable to make the biopsy at our center as the patient had decided to return to her home country for treatment after diagnosis. It was reported that she died 2 months after diagnosis.

## 3 DISCUSSION

Alveolar rhabdomyosarcoma (ARMS) is a malignant neoplasm of the skeletal muscle lineage that is more common in children than in adults, where it has a low incidence and a poor overall prognosis.^1^ True to its aggressive behavior, the presence of a medium of 1‐2 cells undergoing mitosis on 50× oil objective Figure 1I in the bone marrow imprint suggested a very proliferative neoplasm.

Bone marrow infiltration by ARMS is uncommon; some cases can mimic acute leukemia at presentation and thus mislead the diagnosis. We conducted a PubMed search of similar cases of ARMS with a leukemic presentation, finding 41 cases of mimicry reported in the literature, the oldest reported four decades ago.^2^, ^3^, ^4^, ^5^, ^6^, ^7^, ^8^, ^9^, ^10^, ^11^, ^12^, ^13^, ^14^, ^15^, ^16^, ^17^ We added our case and reviewed the data available from these reports on Table 1.

**Table 1:** Summary of cases

Reported cases	42 cases
Rhabdomyosarcoma with initial leukemia presentation	39 cases
Previously known rhabdomyosarcoma	2 cases
Status	27 cases reported dead 15 cases alive or not reported
Not identified primary site	1 case
Adults >18 y	17 cases (40.4%) Dead 13 (76.4%) alive/unknown 4
Children <18 y	25 cases (59.5%) Dead 16 (64%) alive/unknown 9
Lymphoid morphology	13 cases (33.3%)
Undifferentiated Blast leukemia‐like morphology	20 cases (47.6%) 8 unknown (19%)
Blasts positive for periodic acid‐Schiff	6 cases reported
Immunophenotype CD56+/CD45 − Reported useful for diagnosis	19 cases
1st treated as acute leukemia	3 (lymphoblastic leukemia protocol) 2 (myeoblastic leukemia protocol)

The overall mortality of the patients was 64%, and if we take into account that some reports do not provide the final status of the patient the mortality could be higher. It is important to note the lymphoid‐like semblance morphology description in 33.3% of the cases (large cells, round nucleus with lax chromatin, and basophilic cytoplasm with vacuoles); while 47.6% reported undifferentiated/primitive blasts like cells; in 19% they were not specified. Furthermore, in six cases the blasts were reported as periodic acid‐Schiff positive (PAS). ARMS cells are PAS+ which, in the context of lymphoid‐like morphology, can further increase the risk of misdiagnosis. In the cases previously reported five patients were treated as acute leukemia before reaching the final diagnosis.

Immunophenotyping usually shows these cells to be CD56+/CD45− and was useful for the correct diagnosis in 45% of the cases, leading to suspect other neoplasms and wait for the results of the bone marrow Immunocytochemistry before taking further action.

Alveolar rhabdomyosarcoma with leukemic presentation is rare and represents an advanced stage of the disease, with a poor overall survival in both adults and children. Immunocytochemistry and flow cytometry play an important role to avoid misdiagnosis. Our case shares the lymphoid‐like morphology reported in 33% of the cases but the presence of blast conglomerates was a deciding factor to suspect a different neoplasm, which was confirmed thanks to Immunocytochemistry and flow cytometry. To our knowledge, no other cases accompanied by hemophagocytosis and cannibalism have been reported.

The integration of diagnostics tests and techniques in uncommon malignancies is important to avoid a delay in the diagnosis of the patients and secure the best treatment available.

## CONFLICT OF INTEREST

None declared.

## AUTHOR CONTRIBUTIONS

Bernardo López‐Andrade: drafted the manuscript, involved in conception and design, and involved in cytomorphology review. Maria Antonia Duran: revised critically, involved in cytomorphology review. Lourdes Torres and Marta García‐Recio: acquired the clinical data. Laura Lo Riso and Alejandro Formica: acquired the cytomorphology data Rafael F. Ramos: acquired the data and reviewed the bone marrow biopsy. Niurka Cerda: acquired the bone marrow biopsy images and evaluation. Antonia Sampol: revised critically.

## References

[1] van der Graaf W , Orbach D , Judson IR , Ferrari A . Soft tissue sarcomas in adolescents and young adults: a comparison with their paediatric and adult counterparts. Lancet Oncol. 2017;2017(18):e166‐e175. 10.1016/S1470-2045(17)30099-2 28271871

[2] Sandberg AA , Stone JF , Czarnecki L , Cohen JD . Hematologic masquerade of rhabdomyosarcoma. Am J Hematol. 2001;68(1):51‐57. 11559937 10.1002/ajh.1148

[3] Imataki O , et al. Complete mimicry: a case of alveolar rhabdomyosarcoma masquerading as acute leukemia.Diagn Pathol. 2017;12(1):77. 29096655 10.1186/s13000-017-0667-7 PMC5669030

[4] Stall JN , Bailey NG . Metastatic alveolar rhabdomyosarcoma to the bone marrow mimicking acute leukemia. Blood. 2012;120(18):3632. 23281529 10.1182/blood-2012-05-427260

[5] Kern JB , Hii A , Kruse MJ , et al. A leukemic presentation of alveolar rhabdomyosarcoma in a 52‐year‐old woman without an identifiable primary tumor. Int J Surg Pathol. 2015;23(1):75‐77. 25305220 10.1177/1066896914553666 PMC4332771

[6] Reinecke P , et al. Temporary remission of an alveolar rhabdomyosarcoma diagnosed and treated as acute leukemia. Leuk Lymphoma. 2000;36(3‐4):405‐409. 10674913 10.3109/10428190009148862

[7] Yamaguchi K , et al. Alveolar rhabdomyosarcoma of unknown origin mimicking acute leukemia at the initial presentation. Rinsho Ketsueki. 2007;48(4):315‐320. 17515123

[8] Perel Y , Ansoborlo S , Bernard P , Rivel J , Micheau M . Alveolar rhabdomyosarcoma presenting as leukemia. J Pediatr Hematol Oncol. 1998;20(1):94. 9482423 10.1097/00043426-199801000-00018

[9] Chen L , Shah HO , Lin JH . Alveolar rhabdomyosarcoma with concurrent metastases to bone marrow and lymph nodes simulating acute hematologic malignancy. J Pediatr Hematol Oncol. 2004;26(10):696‐697. 15454847 10.1097/01.mph.0000140654.50344.92

[10] Curry S , Bacon CL , Robinson I , Capra M , Smith OP . Leukaemic alveolar rhabdomyosarcoma. Br J Haematol. 2010;151(3):208. 20813011 10.1111/j.1365-2141.2010.08338.x

[11] Jelić‐Puskarić B , Rajković‐Molek K , Raić L , Batinić D , Konja J , Kardum‐Skelin I . Rhabdomyosarcoma with bone marrow infiltration mimicking hematologic neoplasia. Coll Antropol. 2010;34(2):635‐639. 20698143

[12] Naithani R , Kumar R , Mahapatra M , Agrawal N , Saxena R , Sharma S . Pelvic alveolar rhabdomyosarcoma with bone marrow involvement misdiagnosed as acute myeloid leukemia. Pediatr Hematol Oncol. 2007;24(2):153‐155. 17454783 10.1080/08880010601031906

[13] Pérez del Río MJ , et al. Alveolar rhabdomyosarcoma with massive infiltration of the bone marrow as its initial manifestation. Sangre (Barc). 1998;43(3):236‐239. 9741233

[14] Aida Y , Ueki T , Kirihara T , et al. Bone marrow metastasis of rhabdomyosarcoma mimicking acute leukemia: a case report and review of the literature. Intern Med. 2015;54(6):643‐650. 25786457 10.2169/internalmedicine.54.2473

[15] Patiroglu T , Isik B , Unal E , et al. Cranial metastatic alveolar rhabdomyosarcoma mimicking hematological malignancy in an adolescent boy. Childs Nerv Syst. 2014;30(10):1737–1741. 24917491 10.1007/s00381-014-2443-2

[16] Maywald O , Metzgeroth G , Schoch C , et al. Alveolar rhabdomyosarcoma with bone marrow infiltration mimicking haematological neoplasia. Br J Haematol. 2002;119(3):583. 12437629 10.1046/j.1365-2141.2002.03860.x

[17] Chauhan K , Jain M , Shukla P , Grover RK . Rhabdomyosarcoma infiltrating bone marrow. Int J Hematol. 2015;101:1. 25348638 10.1007/s12185-014-1692-x

